# Hemophagocytic lymphohistiocytosis: a rare disease unveiling the diagnosis of EBV-related large B cell lymphoma in a patient with HIV

**DOI:** 10.1007/s12672-022-00476-3

**Published:** 2022-03-21

**Authors:** Alexandra Noveihed, Shiochee Liang, Joel Glotfelty, Ibiyonu Lawrence

**Affiliations:** Department of Internal Medicine, Rutgers-Robert Wood Johnson Medical School, New Brunswick, NJ USA

**Keywords:** Hemophagocytic lymphohistiocytosis, Epstein–Barr virus associated B-cell lymphoma, Acquired immunodeficiency syndrome

## Abstract

**Background:**

Hemophagocytic lymphohistiocytosis (HLH) is a rare disease resulting from the overactivation of the immune system due to under regulation of cytotoxic lymphocytes, macrophages and natural killer (NK) cells. HLH is associated with malignancies, infections, autoimmune disorders and rarely AIDS and is rapidly fatal.

**Case presentation:**

This case report identified a 53 year old male with acquired immunodeficiency syndrome (AIDS) who presented with neutropenic fever of unknown origin. He had two previous hospitalizations prior to the hospitalization diagnosing HLH. The first led to a diagnosis of drug fevers in the setting of treatment for thrombotic thrombocytopenic purpura and subsequent hospitalization led to empiric treatment of hospital acquired pneumonia after workup for intermittent fevers was negative. He was discharged but readmitted 10 days after for recurrence of neutropenic fevers. During this final hospitalization, he was found to have elevated liver enzymes, ferritin, triglycerides and soluble IL-2 receptor with persistent fevers, new splenomegaly and bicytopenia meeting the 2004 HLH criteria. Bone marrow biopsy confirmed the diagnosis of HLH as well as EBV associated large B-cell lymphoma. The patient improved on treatment with steroids, rituximab, tocilizumab, and chemotherapy but ultimately passed away due to refractory septic shock from multi-drug resistant Klebsiella pneumoniae.

**Conclusion:**

This novel case highlights a patient diagnosed with HLH in the setting of several risk factors for the disease, including AIDS, B-cell lymphoma and EBV. Additionally, this case highlights the importance of early consideration of HLH in the setting of neutropenic fever without clear infectious etiology and search for malignancy associated reasons for HLH especially in immunocompromised patients.

## Background

Hemophagocytic lymphohistiocytosis (HLH) is an aggressive, rapidly fatal, and often misdiagnosed hyper-activation of the immune system that can be seen in a variety of malignancies, infections, autoimmune disorders, and rarely HIV [1, 2]. This pathology results in excessive production of cytokines which in turn results in cellular destruction and eventual organ failure [3]. We present the case of a 53-year-old male with HIV/AIDS who was admitted with neutropenic fever in the setting of bone marrow failure, which preceded a diagnosis of HLH from EBV related Large B Cell Lymphoma.

## Case presentation

A 53-year-old African-American male with a past medical history of HIV with CD4 count of 27 and undetectable viral load, hypertension, hyperlipidemia, colitis status post hemicolectomy, polysubstance abuse who presented with fever of unknown origin. Notably, the patient has had two prior admissions three months before this presentation. On his first admission, he was diagnosed with thrombotic thrombocytopenic purpura, responsive to Caplacizumab and prednisone. His hospital course was complicated by intermittent persistent fevers, which was eventually attributed to drug fever. There was consideration of DRESS syndrome during this admission although the patient did not have eosinophilia, which one study suggests is present in 97% of patients with DRESS syndrome [4]. Patient was discharged home but readmitted two weeks later with failure to thrive and was found to have neutropenic fevers of unknown origin. He underwent extensive workup for infectious etiology. CT chest showed persistent stable pulmonary nodules for which bronchoscopy was performed with lavage cytopathology significant only for colonized rare candida spp. He was found to have Epstein–Barr virus viremia. However, with no clear source he was treated empirically for hospital acquired pneumonia. He was discharged after resolution of his fevers to subacute rehab.

**Fig. 1:**
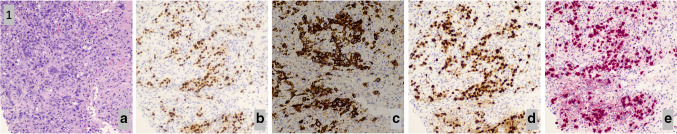
** a**–**e** Retroperitoneal lymph node biopsy. H&E stained core biopsy of the retroperitoneal lymph node shows sheets and clusters of large, pleomorphic cells, with some showing prominent nucleoli. Numerous mitoses are observed. (**a**, H&E). By immunostain, the cells are positive for Pax-5 (**b**), CD30 (**c**), and MUM-1 (**d**). EBER in-situ hybridization is positive (**e**)

**Fig. 2:**
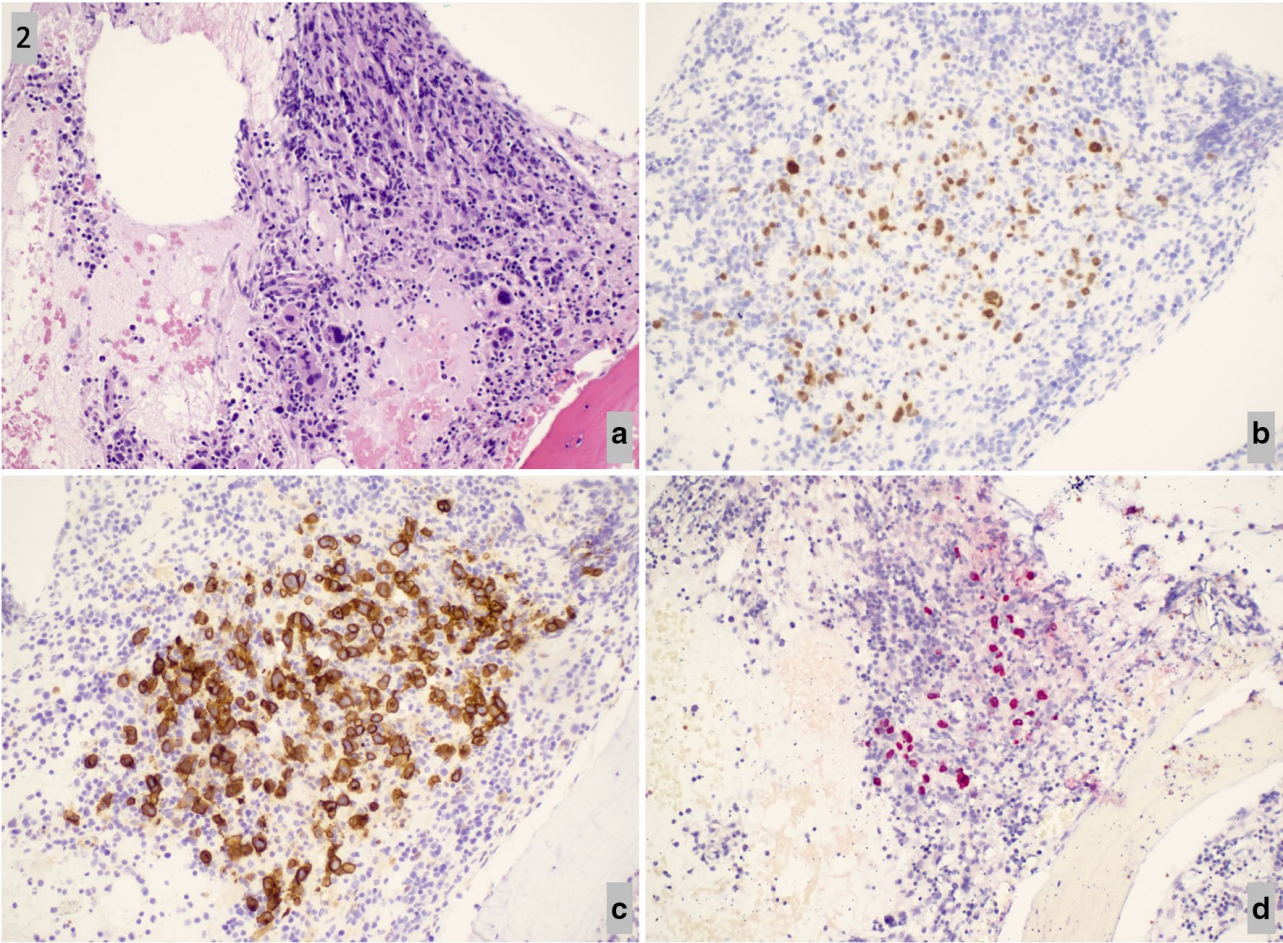
** a**–**d** Bone marrow biopsy. H&E stained bone marrow core biopsy shows a hypercellular bone marrow for age with clusters of large abnormal lymphoid cells (**a**) that are positive for Pax-5 (**b**) and CD30 (**c**) by immunostain, and positive for EBER by in-situ hybridization (**d**)

Ten days after discharge, he was readmitted for recurrence of neutropenic fevers, with absolute neutrophil count 532. Extensive workup revealed persistent fevers, splenomegaly, bicytopenia, elevated LFTs, ferritin, triglycerides, and soluble IL-2 receptor, meeting the 2004 hemophagocytic lymphohistiocytosis (HLH) criteria [5] (Tables 1, 2). Lymph node biopsy confirmed the diagnosis of EBV associated lymphoma (Fig. 1a–e). Bone marrow biopsy confirmed the diagnosis of immunodeficiency associated large B-cell lymphoma with EBV associated HLH (Fig. 2a–d). Patient was treated with a combination of steroids, rituximab, tocilizumab, and chemotherapy. Initially, the patient’s status improved but unfortunately the patient ultimately expired 5 days after intensive care unit (ICU) downgrade due to refractory septic shock secondary to multidrug resistant Klebsiella pneumoniae.

**Table 1:** Diagnostic evaluation of the patient based on 2004 HLH criteria

HLH Criteria	Admission preceding the diagnosis of HLH	On diagnosis of HLH	After initiation of HLH treatment
Fever > 38.5C	Present	Present	Resolved
Splenomegaly	Negative	Present (new)	Decreased in size: 12 cm from 14 cm
Cytopenias (affecting at least 2 of 3 lineages in the peripheral blood) Hemoglobin < 9 g/dL Platelets < 100 × 10^3^/mL Neutrophils < 1 × 10^3^/mL	ANC: 0.4–1.4 Hg: 7.3–8.9 Platelets: 108–220	ANC: 0.5 Hg 7.4 Platelets 88	ANC: 1.90 Hg: 9.4 Platelets 52
Hypertriglyceridemia (fasting > 265 mg/dL) ± Hypofibrinogenemia (< 150 mg/dL)	N/A Fibrinogen: 729–864	TGs 469 Fibrinogen: 197–661	TGs 191
Hemophagocytosis in bone marrow, spleen, lymph nodes, or liver	Negative on bone marrow biopsy	Positive on bone marrow biopsy	N/A
Low or absent NK cell activity	N/A	N/A	N/A
Ferritin > 500 ng/mL	N/A	6751 (on admission) 52,823 (peak)	6226
Elevated sCD25 (soluble IL-2 Receptor)	N/A	5657 U/mL (High)	N/A
CXCL9 (not part of 2004 criteria)	N/A	44,417 pg/mL (High)	N/A

The diagnosis of HLH Disease can be established by 5 of 8 criteria as fulfilled

**Table 2:** Other important biomarkers not included in 2004 HLH criteria but assisting in the diagnosis of HLH

Notable Labs	Admission preceding the HLH diagnosis	Admission with diagnosis of HLH
EBV Viral Titer	38,000	97,900
LDH	225–401	326–3914
ALT/AST	30/14	34/135
Total Bilirubin	0.2	5.6
Direct Bilirubin	< 0.2	4.2
ALP	213	447

## Discussion

HLH is a rare disease associated with overactivation of the immune system, specifically the under regulation of cytotoxic lymphocytes, macrophages and natural killer (NK) cells. While primarily a pediatric entity, cases have been diagnosed in adults, with a predisposition for males, mean age range of 41–67 years, and an estimated incidence rate of 1 per 800,000 people [6, 7]. Etiology is poorly defined but associated with genetic abnormalities specifically impairing perforins (a key delivery molecule for proapoptotic granzymes) [8], especially in the pediatric population. HLH associated with genetic causes is termed primary HLH. Our patient unfortunately cannot be excluded from the diagnosis of primary HLH as genetic causes were not tested during his admission. Nonetheless, our patient had multiple associations to make the likely diagnosis of secondary HLH.

In adults, a 2015 single center study found the etiology of secondary HLH was commonly due to infection (41.1%), followed by malignancies (28.8%). Of the infectious etiologies, HIV and EBV both were the most common causes (5/30 and 5/30) [2]. Viral load of EBV quantified by PCR correlates with disease severity [6]. Interestingly, in our patient, his EBV viral load from previous admission was 38,000. As he further deteriorated and the diagnosis of HLH became clearer, his viral load increased to 97,900 (Table 1). Of the malignancy related causes, B-cell lymphoma was the most common, with 10 out of 21 patients [2]. It is likely that the disease driving the patient’s presentation was a combination of EBV, HIV and large B cell lymphoma.

In our patient, the diagnosis of lymphoma was made on lymph node biopsy (Fig. 1a–e) prior to the pathological diagnosis of HLH seen on bone marrow biopsy (Fig. 2a–d). This diagnosis of lymphoma was EBV-related as evidenced by the positivity of EBER in-situ hybridization on lymph node and bone marrow biopsy. To our knowledge, there have been no retrospective or prospective studies defining the incidence of HLH with underlying HIV, EBV, and B cell lymphoma. We found one case report describing a similar patient presentation [9]. Although NK function and genetics were not performed on our patient, our patient had clear triggers for HLH secondary to immune activation, rather than underlying genetic defects in lymphocytes, thus classifying his case under HLH disease rather than syndrome. Regardless, without treatment, HLH has a high mortality due to multiorgan failure.

**Fig. 3:**
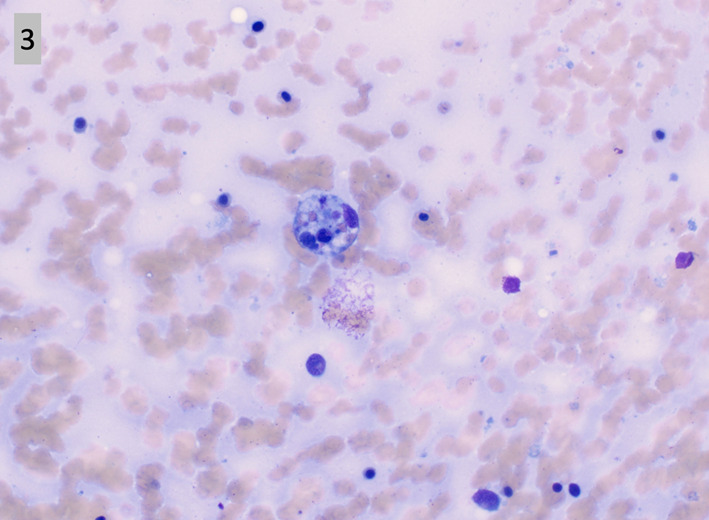
A representative hemophagocytic histocyte is pictured from a Wright–Giemsa stained bone marrow aspirate smear

According to the HLH-2004 trial, which determined the diagnostic criteria for HLH, histological identification of HLH was not required to make a definitive diagnosis. Nonetheless, in patients with clinical suspicion for HLH, bone marrow biopsy to search for hemophagocytosis or underlying malignancy could help aid in the diagnosis [10]. Further complicating the picture, the presence of hemophagocytosis in bone marrow biopsies was neither sensitive nor specific for the diagnosis of HLH. On the admission preceding the admission ultimately diagnosing HLH, our patient had a bone marrow biopsy that was negative for hemophagocytosis. This leads us to question whether bone marrow biopsy should be performed on all patients suspected of HLH [9]. According to a retrospective analysis of HLH confirmed patients, bone marrow biopsy only showed hemophagocytosis in 70% of patients [11]. In our case, bone marrow biopsy showed hemophagocytosis (Fig. 3) while also distinguishing the underlying etiology of HLH (Fig. 2a–d). Our patient case highlights the importance of early consideration of HLH in the setting of neutropenic fever without clear infectious etiology. Additionally, this highlights the importance of identifying the etiology of secondary HLH, including malignancy.

## Data Availability

Data availability is not applicable to this article as no datasets were generated or analyzed during this case report.

## Abbreviations

HLH: Hemophagocytic lymphohistiocytosis
EBV: Ebstein–Barr virus
HIV: Human immunodeficiency virus
AIDS: Acquired immunodeficiency syndrome
